# Potential for rapid antibody detection to identify tuberculous cattle with non-reactive tuberculin skin test results

**DOI:** 10.1186/s12917-017-1085-5

**Published:** 2017-06-07

**Authors:** W. Ray Waters, H. Martin Vordermeier, Shelley Rhodes, Bhagwati Khatri, Mitchell V. Palmer, Mayara F. Maggioli, Tyler C. Thacker, Jeffrey T. Nelson, Bruce V. Thomsen, Suelee Robbe-Austerman, Doris M. Bravo Garcia, Mark A. Schoenbaum, Mark S. Camacho, Jean S. Ray, Javan Esfandiari, Paul Lambotte, Rena Greenwald, Adrian Grandison, Alina Sikar-Gang, Konstantin P. Lyashchenko

**Affiliations:** 1 National Animal Disease Center, Agricultural Research Service, United States Department of Agriculture (USDA), Ames, IA USA; 2 Tuberculosis Research Group, Animal and Plant Health Agency, Addlestone, UK; 3 National Veterinary Services Laboratories, Animal and Plant Health Inspection Service (APHIS), USDA, Ames, IA USA; 4 Veterinary Services (VS), APHIS, USDA, Fort Collins, CO USA; 5 VS, APHIS, USDA, Raleigh, NC USA; 6 VS, APHIS, USDA, East Lansing, MI USA; 7 Chembio Diagnostic Systems, Inc., Medford, NY USA

**Keywords:** Antibody, Bovine tuberculosis, Dual path platform, Multi-antigen print immunoassay, Tuberculin skin test, *Mycobacterium bovis*

## Abstract

**Background:**

Bovine tuberculosis (TB) control programs generally rely on the tuberculin skin test (TST) for ante-mortem detection of *Mycobacterium bovis-*infected cattle.

**Results:**

Present findings demonstrate that a rapid antibody test based on Dual-Path Platform (DPP^®^) technology, when applied 1-3 weeks after TST, detected 9 of 11 and 34 of 52 TST non-reactive yet *M. bovis*-infected cattle from the US and GB, respectively. The specificity of the assay ranged from 98.9% (*n* = 92, US) to 96.0% (*n* = 50, GB) with samples from TB-free herds. Multi-antigen print immunoassay (MAPIA) revealed the presence of antibodies to multiple antigens of *M. bovis* in sera from TST non-reactors diagnosed with TB.

**Conclusions:**

Thus, use of serologic assays in series with TST can identify a significant number of TST non-reactive tuberculous cattle for more efficient removal from TB-affected herds.

## Background

Tuberculosis (TB) in humans and animals may result from exposure to bacilli within the *Mycobacterium tuberculosis* complex such as *M. tb, M. bovis, M. africanum, M. microti, M. caprae, M. orygis, M. suricattae, M. mungi,* or *M. canetti* [1, 2]. *M. bovis* is the species most often isolated from tuberculous cattle. Despite intensive and costly control efforts over many decades, bovine TB persists in most countries adversely affecting animal health, welfare, and trade as well as the livelihoods of producers. Persistence of bovine TB in livestock populations also demands the maintenance of costly federal and regional regulatory networks. Control strategies rely largely on ante-mortem testing and slaughter inspection to identify livestock herds at risk. With cattle, the principal ante-mortem tests for presumptive diagnosis of bovine TB are immunoassays that detect cell-mediated responses, including both in vivo [i.e., tuberculin skin test (TST)] and in vitro [i.e., interferon gamma release assay (IGRA)] methods [3–5]. In many countries, TST is applied as the primary test and IGRA may be used as an ancillary test in cattle to maximize the number of infected animals identified or as a confirmatory test [6]. The most common applications for ante-mortem testing include routine surveillance to identify *M. bovis*-infected herds, test and removal schemes, movement tests, epidemiologic trace-back testing, and in TB-affected herds to delineate animals going to a slaughter plant versus being condemned for rendering. While used extensively for over 100 years in cattle, the TST does have a number of severe shortcomings. The sensitivity of TST ranges broadly from 55 to 97% depending on the type and technical variations of test applied, quality of purified protein derivative (PPD), environmental exposure/burden to atypical mycobacteria, and many other factors [4, 5]. Thus, improved ante-mortem tests and/or testing strategies for bovine TB are greatly needed.

Over the past decade, a new generation of serologic tests designed to detect antibodies to multiple *M. bovis* specific antigens have emerged for application in cattle [7–13]. Of these, an ELISA using MPB83 and MPB70 antigens (*M. bovis* Ab Test, IDEXX Laboratories, Westbrook, Maine, US [10]) is approved by the *Office International des Epizooties* and US Department of Agriculture for discretionary use in cattle; however, application of this test has been limited primarily to confirmation of infection. Injection of PPDs for TST significantly boosts antibody responses in *M. bovis* infected cattle, including animals without prior detectable antibody responses [10, 11, 14–16]. The enhanced IgG responses are elicited by *M. bovis* specific antigens (e.g., MPB83 and MPB70) and characterized by accelerated antibody affinity maturation [17]. The boosted antibody responses wane beginning ~1-2 months after PPD administration; however, they can be further increased upon PPD re-injection [17]. Despite these advances, existing antibody assays generally lack diagnostic sensitivity, especially in early infection, and thus require further improvements [4–6].

In the present study, sera from *M. bovis*-infected cattle identified as TST non-reactors in naturally-exposed herds within the US (*n* = 11) and GB (*n* = 52) were evaluated for antibody responses to *M. bovis* specific proteins using a next generation immunochromatographic test based on Dual-Path Platform (DPP^®^) technology developed by Chembio Diagnostic Systems, Inc. (Medford, New York, US) [9]. Findings demonstrate the potential for use of antibody assays to detect *M. bovis* infection in TST non-reactive cattle within TB-affected herds.

## Methods

### Naturally-infected herds

#### Great Britain

Sera (*n* = 127) from GB were obtained from *M. bovis-*infected cattle detected during routine surveillance, including multiple herds and animals of diverse age, gender, breed, and management systems. All animals received a single intradermal comparative cervical test (SICCT) and of these, 52 animals were SICCT negative, IGRA positive (blood collected for IGRA and serum ~60 days post-SICCT) with tuberculous lesions detected postmortem. The other 75 animals were SICCT positive and not tested by IGRA (serum collection circa 3 weeks post-SICCT at the abattoir) with tuberculous lesions upon postmortem and *M. bovis* isolated from lesions*.* Serum samples were also collected from 50 SICCT negative, IGRA negative cattle located in a TB-free region of GB.

#### Texas, US

A cow with tuberculous lesions (later confirmed as *M. bovis* upon mycobacterial culture) was detected upon routine inspection at an abattoir in 2014. The source herd of this cow was determined to consist of approximately 11,000 Holstein dairy cattle. On postmortem examination of initial caudal-fold tuberculin test (CFT) reactors, a tuberculous lesion rate (confirmed by histopathology) of 1.5% was found. Based on epidemiologic risk factors, it was determined by regulatory officials and dairy management that destocking the dairy would be the best approach to rid it of *M. bovis*. A CFT and collection of blood for serological testing was completed 10-15 days prior to postmortem examination at destocking. A 2% overall prevalence of TB (visible tuberculous lesions confirmed by histopathology) was noted in cattle among CFT positive (35%) and CFT negative cattle (0.4%). From the CFT false-negative cattle, 7 serum samples obtained within 3 weeks after PPD injection were available for serologic analysis.

#### Michigan, US

Two cattle herds, one beef and the other dairy (mostly Jersey), within the TB-endemic region of Michigan (Northeast corner of the lower peninsula) were identified as TB-affected via routine surveillance in 2015. Animals within these two herds had received CFTs yearly for TB surveillance prior to 2015. Upon identification of tuberculous animals in 2015, CFTs were applied more frequently (~4-6 month interval) in both herds. Based on the presence of gross lesions upon depopulation, prevalence of TB was estimated to be ~21% (17/81) and 9% (53/561) for the beef and dairy herds, respectively. Four serum samples collected from *M. bovis*-infected CFT non-reactors within 3 weeks after the last CFT administration were available for serologic testing.

### Multi-antigen print immunoassay (MAPIA)

MAPIA was performed as described previously [18]. Briefly, a panel of ten *M. tuberculosis*-complex antigens immobilized on nitrocellulose membrane included ESAT-6 (Rv3875), CFP10 (Rv3874), MPB64 (Rv1980c), MPB70 (Rv2875), MPB83 (Rv2873), CFP10/ ESAT-6 fusion protein, MPB70/MPB83 fusion protein, MPB70/CFP10/Rv0934 fusion protein, bovine PPD (bPPD), and *M. bovis* culture filtrate (MBCF). Strips were cut and blocked with 1% nonfat milk in PBS with 0.05% Tween 20 for 1 h prior to incubation with serum samples diluted 1:40 in blocking solution for 1 h. After washing, strips were incubated with peroxidase-conjugated Protein G (Sigma, St. Louis, MO) diluted 1:1000 for 1 h, washed, and developed with 3,3′,5,5′-tetramethyl benzidine (TMB) (Kirkegaard & Perry Laboratories, Inc., Gaithersburg, MD).

### Dual-path platform (DPP^®^) assay

Bovine IgG and IgM antibodies to CFP10/ESAT-6 and MPB70/MPB83 protein fusions were detected as described previously [17] using goat anti-bovine IgG and anti-bovine IgM antibodies (Kirkegaard & Perry Laboratories Inc.) conjugated to colloidal gold nanoparticles by Chembio standard procedure. Sera were diluted 1:20 in sample buffer for testing by DPP assay and results were recorded at 15 min using an optical reader to measure test band reflectance in relative light units (RLU), as previously described [17]. Using pre-established cut-off values of 20 RLU for CFP10/ESAT-6 antigen and 40 RLU for MPB70/MPB83 antigen (same for both IgM and IgG antibody detection), DPP assay readouts were expressed as signal-to-cutoff ratios, with any values ≥1.00 being interpreted as a reactive result and any value <1.00 being considered as a non-reactive result.

### Data analysis

Diagnostic performance of the serologic assays was assessed against the gold standard of *M. bovis* culture and/or IS-6110 PCR by calculating test sensitivity and specificity using available software [19] and presented with the 95% confidence intervals (CI). Fisher’s exact test was used for analysis of antigen recognition by bovine antibodies in the present study.

## Results

### Antibody detection in TST non-reactors versus TST reactors: Samples from Great Britain

With sera from *M. bovis* infected cattle in GB, the overall IgG reactivity rates in the DPP assay were similar for SICCT reactors (60%, *n* = 75) and non-reactors (65.4%, *n* = 52). The DPP assay specificity assessed with sera from 50 SICCT negative, IGRA negative cattle from TB non-endemic regions of GB was 96% (Table 1). Of note, the response rate by *M. bovis*-infected cattle to each fusion antigen differed significantly (*p* < 0.01, Fisher’s exact test) based on TST reactivity. The ratio of MPB70/MPB83 to CFP10/ESAT-6 reactivity was approximately 3.6:1 for TST reactors versus 1:1 for TST non-reactors (Table 1). In line with the above, only 1 of 75 TST reactors but 13 of 52 TST non-reactors produced antibody solely to CFP10/ESAT-6 yet not to MPB70/MPB83. Thus, the immunodominance of serologically related MPB70 and/or MPB83 proteins typically detected in *M. bovis* infected cattle after PPD administration for SICCT was less evident with TST non-reactors, as reactivity rates to CFP10/ESAT-6 and MPB70/MPB83 were essentially equivalent, and there were considerably more CFP10/ESAT-6 antibody responders within the TST non-reactor subset. This observation demonstrates the potential value for use of additional antigens to maximize the sensitivity of serologic tests, particularly with TST non-reactors.

**Table 1:** IgG reactivity rates in tuberculin skin test reactors and non-reactors found among *M. bovis*-infected cattle in GB

Animal group^a^	No. of animals	Individual antigen reactivity rates in DPP assay^b^		DPP assay reactivity rate^b^ (%, 95% CI)
		CFP10/ESAT-6	MPB70/MPB83	
*M. bovis* infected, TST Reactors	75	12 (16%)	43 (57.3%)	45 (60%; 95% CI: 48,71.2)
*M. bovis* infected, TST Non-Reactors	52	21 (40.4%)	21 (40.4%)	34 (65.4%, 95% CI: 50.9, 78)
Total *M. bovis* infected	127	33 (26%)	64 (50.4%)	79 (62.2%, 95% CI: 53.2, 70.6)
TB-free, TST Non-Reactors	50	2 (4%)	2 (4%)	2 (4%, 95% CI: 0.5, 13.7)

^a^Sera obtained from animals in multiple herds and of diverse age, gender, breed and management systems

^b^Data are presented as number (percent) positive per group for each antigen or assay

### Antibody detection in TST non-reactors: Opportunistic samples from the United States

During 2015 – 2016, two herds from disparate regions of the US were under-going whole herd depopulation due to *M. bovis* infection within the herds. Antemortem testing was used to delineate animals going to a slaughter plant (test negative) versus being condemned for rendering (test positive). Serum samples were available for serologic analysis from 11 CFT non-reactive adult cows with gross tuberculous lesions from the two herds [Texas (*n* = 7) and Michigan (*n* = 4)]. Infection was confirmed in these animals by histopathology, mycobacterial culture, and/or IS-6110 PCR (Table 2). Each of the animals had received a CFT ~6 months prior to the non-reactive CFT; of which 4/7 (Texas) and 0/4 (Michigan) were reactive at that earlier time point, thus indicating TST reversion in four of the animals. Serum samples collected ~1-3 weeks after the last CFT were tested with the DPP assay for the presence of IgM and IgG antibodies to CFP10/ESAT-6 and MPB70/MPB83. Only 4/11 animals had IgM to MPB70/MPB83, whereas none produced IgM to CFP10/ESAT-6. In contrast, relatively potent IgG responses were elicited by MPB70/MPB83 and CFP10/ESAT-6 in 8/11 and 3/11 animals, respectively (Table 3). IgM readouts were generally lower than those obtained for IgG, suggesting little added value of IgM antibody detection from a serodiagnostic sensitivity perspective. The one exception was with animal #364 which had a high IgM and a borderline IgG signal (Table 3). Development of transient IgM responses early in the course of experimental *M. bovis* infection, as well as shortly after TST administration, has been demonstrated in previous studies using different strains of *M. bovis* [11, 17, 20]. Thus, the present findings in the US demonstrated antibody responses to *M. bovis* antigens in ~82% of TST non-reactive cattle with confirmed TB. The specificity of the DPP assay evaluated on 92 samples from TB-free states within the US was 98.9% (95% CI: 94.1, 99.9) or somewhat higher than that found in GB (96.0%; 95% CI: 86.3, 99.5), although not statistically significant (*p* = 0.3).

**Table 2:** Diagnostic characterization of TST false-negative cattle identified in US herds infected with *M. bovis*

State	Animal ID #	CFT^a^ results		Postmortem examination results			
		Jul-2015	Jan-2016	Gross lesions	Histopathology	PCR^b^	Culture
TX	755	Pos	Neg	Present	TB compatible^c^, IS6110 Pos^d^	Neg	Neg
	272	Pos	Neg	Present	TB compatible	Pos	ND^e^
	976	Neg	Neg	Present	TB compatible	Pos	ND
	857	Pos	Neg	Present	TB compatible	Pos	ND
	676	Pos	Neg	Present	TB compatible	Pos	ND
	889	Pos	Neg	Present	TB compatible	Pos	ND
	352	Neg	Neg	Present	TB compatible	Pos	ND
MI^f^		Sep-2015	Jan-2016				
	855	Neg	Neg	Present	TB compatible	ND	*M. bovis*
	370	Neg	Neg	Present	TB compatible	ND	*M. bovis*
	364	Neg	Neg	Present	Not compatible	ND	*M. bovis*
		May-2015	Oct-2015				
	809	Neg	Neg	Present	TB compatible	ND	*M. bovis*

^a^CFT, caudal fold test (i.e., intradermal *M. bovis* PPD injected into the caudal skin fold and response determined by palpation 72 h after injection) was applied <3 wks prior to collection of serum

^b^PCR, polymerase chain reaction for either *M. tb* complex IS6110 or 1081 DNA on fresh tissue

^c^Microscopic granulomatous lesions consistent with bovine TB and containing acid-fast bacilli

^d^IS6110 DNA by polymerase chain reaction on formalin-fixed tissue

^e^ND, not done

^f^Animals # 855, 370, and 364 were from a beef herd while #809 was from a dairy herd

**Table 3:** Quantitative measure of IgM and IgG responses produced by TST false-negative cattle in the US

Animal ID #	Reactivity in the DPP assay^a^			
	CFP10/ESAT-6		MPB70/MPB83	
	IgM	IgG	IgM	IgG
755	0	0.35	0	**6.15**
272	0	0	**1.15**	**1.08**
976	0	**1.50**	0.75	**7.45**
857	0	0	0.15	0
676	0	0	**1.48**	**4.85**
889	0	**2.05**	0	**19.15**
352	0	0.30	0	**5.00**
855	0	0	0	0
370	0	0	**3.10**	**14.95**
364	0	0.50	**6.78**	**1.58**
809	0	**14.45**	0.55	**6.23**

^a^Data are presented as signal-to-cutoff ratios with ≥1.00 considered as antibody reactive results (shown in bold)

### Antigen recognition by IgG antibodies in sera from TST non-responders

**Fig. 1:**
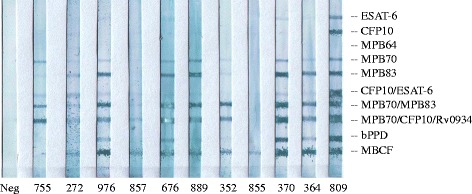
MAPIA testing of TST non-reactive cattle diagnosed with *M. bovis* infection in the US. Assay was performed as described in Methods. Antigens printed onto nitrocellulose membrane are shown on the right. Results are presented for sera from a negative control (on the left), 7 animals from TX, and 4 animals from MI. Animal ID numbers are shown on the bottom (see Table 2 for diagnostic characterization). Visible bands on the strips indicate the presence of IgG to corresponding antigen(s). Intensity of the bands generally correlates with the antibody level

Serum samples from the US cattle were also analyzed by MAPIA to determine antigen recognition patterns. Sera from 10 of 11 cattle reacted with multiple recombinant antigens of *M. bovis* (Fig. 1). The one animal (#855) which had a negative result in the DPP assay, exhibited IgG binding only to MBCF in MAPIA. Animal #857, which was the second DPP non-reactor in this group (Table 3), displayed in MAPIA weak reactivity with the two fusion antigens containing MPB70. Based on line intensity, the magnitude of antibody responses and antigen recognition profiles varied among the animals. The most reactive antigens included MPB70 and MPB83 proteins, MPB70/MPB83 and MPB70/CFP10/Rv0934 hybrids, as well as bPPD and MBCF. For *M. bovis*-infected TST non-reactors, the role of CFP10/ESAT-6 in eliciting antibody responses was not significant in the US (Table 3, Fig. 1) as compared to the GB set of specimens (Table 1). Overall, the antigen recognition results supported the immunodominance of MPB70 and MPB83 proteins in *M. bovis* infection of cattle [8, 11, 17, 20].

## Discussion

A major impediment to the control of bovine TB is the relatively poor accuracy of current ante-mortem tests compounded by difficulties in reliably detecting tuberculous lesions and/or the agent in all infected animals upon slaughter surveillance. TST non-reactive cattle are particularly problematic when applying test and remove strategies in TB-affected herds. According to Lepper et al. [21], “Anergy to tuberculin is defined as the failure of an animal with visible evidence of tuberculosis to show a palpable cutaneous delayed hypersensitivity response to a tuberculin, at the time when the test is read.” Such TST non-reactive animals, however, may still be responsive on other cell-mediated (e.g., IGRAs [22, 23]) or antibody detection immunoassays [24, 25]. In general, TST non-responsiveness is more common in animals from herds with high within-herd prevalence and in animals at advanced stages of disease [21, 22]. Desensitization as a result of repeated short interval application of TSTs may also lead to reduced TST responses associated with increased interleukin-10 (IL-10) and decreased IL-1β production to TB antigens [26]. Our findings further demonstrate that certain *M. bovis*-infected cattle can escape detection by TST by reverting from reactors to non-reactors within several months; however, a significant proportion of these animals can be identified by ancillary tests, such as the antibody tests described herein or IGRA [27].

In the present study, *M. bovis* specific antibody was detected by the DPP assay in ~82% and ~65% of *M. bovis-*infected, TST non-reactive cattle from the US and GB, respectively. As reported previously, an in-house MPB83-based ELISA detected 9 of 20 (45%) SICCT non-reactors diagnosed with bovine TB in GB [28]. Similarly, the MPB70/83 antibody reactivity rate in the group of TST non-reactive cattle in GB was ~40% as demonstrated by DPP assay (for MPB70/MPB83 antigen test line only, present study) and also shown independently by a commercial ELISA (*M. bovis* Ab Test, IDEXX Laboratories, Westbrook, Maine) using a cocktail of MPB70 and MPB83 proteins (unpublished data). Importantly, integrating CFP10/ESAT-6 to supplement MPB70/MPB83 antigen in the DPP assay enhanced the overall test sensitivity to ~65% in TST non-reactive cattle in GB, thereby highlighting the added benefit of combining multiple antigens in serologic assays.

## Conclusions

The present findings demonstrate the potential for use of antibody tests in TB-affected herds to rapidly identify *M. bovis*-infected, but TST non-reactive cattle. The DPP assay may also be considered for use in series with TST as a movement test, particularly as the assay may be applied pen-side without the need for laboratory equipment and results are available within 20 min. For example, Mexican cattle are required to have a negative CFT within 60 days of entry into the US. Thus, the DPP assay could be applied after CFT in Mexico or at the US/Mexico border as a further safeguard against entry of *M. bovis*-infected cattle into the US. For this application, given a very low disease prevalence in the large number of cattle crossing the US border with recent CFT negative results, a viable serologic assay would need to have an extremely high specificity (>99.9%) to provide an acceptable positive predictive value at a low pre-test probability. In the case of the DPP assay, the target specificity can be established and validated by having a cut-off value adjusted to meet this key requirement without a significant loss of diagnostic sensitivity. Given the limited number of samples available for the study, present findings should be considered preliminary and more extensive studies particularly in other bovine TB endemic countries are warranted to further verify the utility of this approach. These studies should also include sera from cattle infected with non-tuberculous mycobacteria (e.g., *M. avium* subsp. *paratuberculosis*, *M. kansasii,* etc.) to determine the possible interaction of these mycobacteria on this approach, particularly as injection of PPDs for SICCT in cattle vaccinated with heat inactivated Johne’s disease vaccine may induce false positive responses to MPB83/70-based antibody assays [29]. With that said, present findings clearly demonstrate that use of serologic assays in series with TST can identify a significant number of TST non-reactive tuberculous cattle for more efficient removal from TB-affected herds.

## Abbreviations

CFP10: Culture filtrate protein 10 kDa protein
CFT: Caudal fold test
CI: Confidence interval
DPP: Dual path platform
ESAT-6: Early secretory antigenic target 6 kDa protein
GB: Great Britain
IGRA: Interferon-γ release assay
MAPIA: Multi-antigen print immunoassay
MPB70: Mobility protein of bovis, 70
MPB83: Mobility protein of bovis, 83
PPD: Purified protein derivative
RLU: Relative light units
SICCT: Single intradermal comparative cervical test
TB: Tuberculosis
TST: Tuberculin skin test
US: United States
